# Repurposing cephalosporins as excellent anticancer agents and chemosensitizers for inflammation-driven cancer therapy

**DOI:** 10.1038/s41598-025-25287-8

**Published:** 2025-11-21

**Authors:** Nianqiu Liu, Weihan Cao, Jiefu Tang, Ruimei Dong, Juan Guo, Zhuang Luo, Qian Yao, Song Teng, Zhuoxuan Liang, Yuntao Yang, Menying Gu, Jie Zhou, Wenlin Chen, Hongmin Liang, Xiaoqiong He

**Affiliations:** 1 The School of Public Health, Kunming Medical University, Kunming, Yunnan China; 2 The Key Laboratory of Breast Surgery, The Third Affiliated Hospital of Kunming Medical University, Yunnan Cancer Hospital, Kunming, Yunnan China; 3 The Department of Ultrasound, The First Affiliated Hospital of Kunming Medical University, Kunming, Yunnan China; 4 The Department of Pulmonary and Critical Care Medicine, The First Affiliated Hospital of Kunming Medical University, Kunming, Yunnan China; 5 The Institute of Yunnan Tumor, The Third Affiliated Hospital of Kunming Medical University, Kunming, Yunnan China

**Keywords:** Cephalosporin antibiotics, Chemical structure anticancer activity, Inflammation driven carcinogenesis, Chemotherapy sensitization, HMOX1, Cancer therapy, Tumour biomarkers

## Abstract

Could cephalosporin antibiotics, extending beyond their established antimicrobial role, be repurposed as precision anticancer agents and chemosensitizers, particularly against inflammation-driven carcinogenesis? To address this question, this study systematically evaluated the anti-colorectal cancer efficacy of cephalosporins both as monotherapies and in synergistic combinations, elucidating their underlying molecular mechanisms. Employing combinatorial phenotypic screening (viability, cell cycle, apoptosis, colony formation), BALB/c nude mouse xenografts, and omics profiling (RNA-seq, RT-qPCR), we identified conserved anticancer pathways and core regulatory axes. Among 18 evaluated cephalosporins, therapeutic specificity was largely associated exclusively variable side-chain moieties, not the conserved β-lactam core. Cefamandole nafate (CAN) and cefuroxime sodium (CUS) demonstrated potent dual efficacy against colorectal cancer model while enhancing cisplatin chemosensitivity. Building on links to inflammation-driven chemosensitization, CUS synergistically potentiated cisplatin and levofloxacin cytotoxicity in colorectal cancer. This synergy was mechanistically driven by apoptosis induction, cell cycle arrest, significant up-regulation of HMOX1 (80-fold peak in combinations; 40-fold as monotherapy) and DDIT3, coupled with down-regulation of MUC1, CASC19, KRT23, SPNS3, LFNG, HS3ST1, NCOA5, and GJB4. Crucially, we reveal for the first time that CUS significantly up-regulates HMOX1 expression in HCT116 cells in a dose-dependent manner, establishing this ferroptosis regulator as the central effector governing both intrinsic anticancer activity and chemosensitization. This study unveils the translational potential of repurposing cephalosporins for combinatorial precision oncology strategies targeting inflammation-driven cancers.

**Supplementary Information:**

The online version contains supplementary material available at 10.1038/s41598-025-25287-8.

## Introduction

Cancer remains a global health crisis, ranking as the second leading cause of mortality worldwide. In 2020 alone, 19.3 million new cancer cases and 10.0 million cancer-related deaths were reported globally^1^, with China accounting for 4.57 million incident cases and 3.0 million fatalities. Projections indicate 1,918,030 new diagnoses and 609,360 deaths in the United States during 2022^2^. While chemotherapy remains a cornerstone of cancer management, its utility is constrained by systemic toxicity and acquired multidrug resistance^2^, underscoring the urgent need for safer, more effective therapeutic strategies. Drug repositioning has emerged as a cost-effective paradigm to accelerate anticancer discovery, leveraging existing pharmacotherapies with established safety profiles^3,4^.

The inflammation-cancer nexus is well-established, with chronic inflammatory microenvironments driving tumor initiation, progression, and therapeutic evasion^5^. Mounting evidence positions inflammatory signaling as a universal catalyst across diverse malignancies^6^, prompting interest in dual-purpose agents that simultaneously suppress carcinogenesis and potentiate conventional therapies^7^. Intriguingly, certain antibiotics—including β-lactams and fluoroquinolones—exhibit off-label anticancer properties beyond their antimicrobial roles^8,9^. Our prior work identified cephalosporins as off-label therapeutics for nasopharyngeal carcinoma (NPC)^10^, while levofloxacin demonstrated broad-spectrum tumoricidal activity^11^. Notably, cefotaxime and levofloxacin synergized with standard chemotherapeutics in NPC models, enhancing efficacy while mitigating resistance^12,13^.

Despite these advances, the structural determinants underlying cephalosporins’ heterogeneous anticancer activity remain enigmatic. While all share a conserved β-lactam backbone, their therapeutic potency varies dramatically across cancer types—a paradox exemplified by our preliminary screen of five cephalosporins (second- and third-generation) showing lineage-specific efficacy^10–13^. This disparity raises critical questions: What structural features govern cephalosporins’ tumor-selective activity? Can their shared core be engineered to optimize chemo-sensitization?

This study systematically maps the structure–activity relationships of 18 cephalosporins across histologically diverse malignancies, combining phenotypic screening with mechanistic dissection to resolve two fundamental unknowns: The chemical moieties dictating β-lactam antibiotics’ anticancer specificity; the common pathway through what enhance their anti-cancer activity.

## Materials and methods

### Cephalosporin antibiotics

18 commonly used cephalosporin antibiotics, cefamandole nafate (CAN), cefuroxime sodium (CUS), cefepime hydrochloride (CPH), cefotaxime sodium (COS), Layangtoubaona (LYT), cefmetazole sodium (CMS), cefathiamidine (CAM), ceftriaxone sodium (CTS), cefmenoxime hydrochloride (CMH), ceftazidime (CTD), cefoxitin sodium (CXS), Cefminox Sodium (CNS), Cefpiramide sodium (CIS), Cefazolin Sodium (CAS), Cefazolin Sodium Pentahydrate (CASP), Ceftezole sodium (CES), Ceftizoxime Sodium (CZS), and Cefodizime sodium (CDS) injections were purchased from hospitals in Kunming and freshly dissolved into neutral saline before use (stored in the dark at 4 °C). The recommended maximum dose of 5 cephalosporine antibiotics (CAN, COS, CTD, CXS and CZS) used in the anti-infection treatment is 12 g/day in an adult (50–60 kg body weight (bw)) equating to 200–240 µg/g bw (200–240 mg/kg bw). Referring to our previous study^10^, the final concentrations of all cephalosporin antibiotics were 62.5, 125, 250, 500 and 1000 μM/L (similar to 25, 50, 100, 200 and 400 µg/mL in COS^10^) for in vitro cell culture study. CUS was dissolved in neutral saline and the doses used in mice (ip) was 100, 200 and 300 mg/kg bw.

### Cell culture

16 human cancer cell lines were studied, including 6 nasopharyngeal carcinoma cell lines with different histological characters (CNE1, CNE2, 5-8F, TW03, HONE1, and C666-1), 3 lung carcinoma cell lines (A549, H460 and XWLC05), cervical carcinoma (Hela), hepatocellular carcinoma (HepG2), bladder cancer (T24), colorectal carcinoma (HCT116), pancreatic cancer (PANC1), neuroglioma (U251) and breast carcinoma (MDA-MB231). These cell lines were kindly provided by Institute of Yunnan Tumor stocks, or professor Zhou from Guangxi Medical University stocks, or purchased from the Cell Bank of Kunming Animal Institute, Chinese Academy of Science (Kunming, China). All cells (passage number 1–20) were cultured in fresh DMEM/F12 medium (HyClone) supplemented with 10% fetal bovine serum (FBS) (HyClone) and 1% P/S (HyClone) at 37 °C in a 5% CO_2_, humidified incubator.

### Mice

6–8-week-old male balb/c nude mice (15–20 g) were purchased from Beijing Vital River Laboratory Animal Technology Co., Ltd (Beijing, China). Mice were housed in the temperature- and humidity-controlled Specific Pathogen Free Animal Facility at Kunming Medical University, with a 12-h light–dark cycle. Mice were fed autoclaved distilled water and autoclaved rodent chow. All animal experiments were approved by the Experimental Animal Department of Kunming Medical University (approval number: kmmu2021305), and all methods were performed in accordance with relevant guidelines and regulations. The euthanasia procedures for mice followed ARRIVE guidelines^14^, Place the mouse in its cages and expose it to CO₂ gas at a flow rate that displaces 70% of the cage volume per minute. The animal will become deeply narcotic and eventually die.

### Cell viability assay

Cell viability was determined by the MTT method. Cells were seeded into 96-well plates at a density of 4000 cells per well in 200 µl complete DMEM/F12 medium and incubated overnight. The following day, the original medium was removed and replaced with 200 µl fresh DMEM/F12 medium (supplemented with 10%, free of P/S) containing different concentrations of samples. For each group, 8 replicate wells were treated with a given drug concentration at the same time. Cells were then incubated for 72 h. After removing the medium, 200 µl new complete medium containing 10% MTT (5 mg/ml) was added into each well. Plates were incubated for another 4 h. After carefully removing the MTT medium, 150 µl DMSO was added into each well. Plates were then shaken in the dark for 5 min. Optical density (OD) values were determined by a microplate reader at 490 nm. The highest and the lowest OD values for each set of replicates were removed and the remaining 6 OD values in each group were retained for statistical analysis. Two or three independent experiments were done for some cancer cell lines (CNE2, 5-8F, HONE1, HepG2, HCT116, PANC1, H460 and MDA-MB231). Inhibition rates of cell viability were calculated using the following formula:

$${\text{Inhibition rate }} = \, \left( {{\text{Ac }} - {\text{ Ad}}} \right) \, /{\text{ Ac }} \times { 1}00\% .$$

Ac: the corrected absorbance (OD value) of the vehicle control group.

Ad: the corrected absorbance (OD value) of the drug group.

Corrected absorbance = OD value of sample group − OD value of blank control group.

### Clonogenic colony formation assay

800 HCT116 cells or 400 5–8F cells were seeded into each well of 6-well culture plates and allowed to adhere overnight in the incubator. The next day, media was replaced with 2 ml of fresh medium (free of P/S) containing different concentrations of drugs. Cells were treated with CUS (62.5, 125 and 250 μM/l in 5–8F; or 125, 250 and 500 μM/l in HCT116), CUS + DDP (DDP: 0.5 μg/ml) and CUS + LH (LH: 25 μg/ml) for 14 days respectively. 3 replicates wells were done in each group each time with 3 duplicates. During the period, medium was renewed on the 3th, 6th, 9th and 12th day while checking colony and cell morphology. Media was gently removed on the 14th day, and each well was washed once with 2 ml PBS, fixed with 5% paraformaldehyde for 15 min. Wells were then washed with 2 ml PBS, stained with 0.5 ml 0.5% crystal violet for 10 min, and washed again with 2 ml PBS before acquiring pictures with a camera and counting the number of colonies. The inhibition rate was calculated by:

$${\text{Inhibition rate }} = \, \left( {{\text{Nc }} - {\text{ Nd}}} \right) \, /{\text{ Nc }} \times { 1}00\% .$$

Nc: the clone number of the vehicle control group.

Nd: the clone number of the drug group.

### Detection of cell cycle and apoptosis by flow cytometer

HCT116 and 5–8F cells were treated for 48 h with neutral saline (NC group), 200, 300, or 400 μM/l CUS respectively. For the detection of cell cycle position, cells were collected after 48 h of drug treatment and slowly resuspended into 5 ml of pre-cooling 70% ethanol and fixed overnight at 4 °C, and then centrifuged at 1000 rpm for 5 min. The fixative was discarded and cells were rinsed twice with 2 ml PBS. Cells were resuspended with 500 μl of PI/RNase staining solution and stained for 15 min at room temperature in the dark. At least 10,000 cells in each sample were analyzed by flow cytometry.

An Annexin-V FITC/PI kit (7sea biotech Co., Ltd) was used for apoptosis examination. Cells were treated as for cell cycle analysis. All adherent and floating cells were collected and treated according to the protocol of the kit. Cells were resuspended in 0.4 ml binding buffer before adding 5 μl Annexin-V FITC and incubating on ice for 15 min. Then, 10 μl PI was added to each sample and incubated in the dark for 30 min at room temperature prior to flow cytometry (> 10,000 cells/sample). Differential Annexin-V FITC and PI staining patterns were used to indicate cell populations that were viable (Annexin-V FITC negative, PI negative), early apoptotic (Annexin-V FITC positive but PI negative), late apoptotic (positive for both Annexin-V FITC and PI), and necrotic and mechanically damaged (Annexin-V FITC negative but PI positive). Three biological replicates were performed for the cell cycle and apoptosis experiments.

### Tumor growth inhibition in HCT116 xenograft model

Colorectal carcinoma HCT116 cells were collected and rinsed with PBS twice and resuspended to a density of 1 × 10^8^ cells/ml using fresh and cooled DMEM/F12 medium (free of FBS and P/S). Each mouse was inoculated subcutaneously with a 0.1 ml cell suspension on the right-side flank. Tumor-bearing mice were used for in vivo anticancer study when the average tumor volume reached 100 ~ 200 mm^3^. Tumor-bearing mice were randomly allocated into 6 groups (7 tumor-bearing mice in each group) according to tumor volume: a negative control group (NC, neutral saline), three CUS treating groups (CUS1, CUS2, and CUS3; 100, 200, and 300 mg/kg.bw respectively), a DDP positive control group (2 mg/kg bw), and a combination group (CUS2 + DDP). Mice were injected CUS intraperitoneally once a day on the morning (9:00 a.m.) using a volume of 0.10 ml/10 g.bw per injection. DDP was injected once a day in the morning at one-day interval. The body weight and tumor volume of each mouse were measured every 4 days. Mice were anesthetized by intraperitoneal injection of 1% concentration of pentobarbital sodium on the 16th day of drug treatment. Tumors were carefully isolated and weighed. An auto-reading caliper was used to measure the size of the tumor by the same operator throughout the study. Tumor volume was calculated using the following formula.

Tumor volume (TV) = (a × b^2^)/2, “a” is the longitude diameter; “b” is the short diameter.

Relative tumor volume (RTV) = V_T_/V_0_, V_0_ is the initial tumor volume and V_T_ is the tumor volume measured at each examination point.

Relative proliferation ratio (RPR) = (T_RTV_/C_RTV_) × 100%, T_RTV_ is the relative tumor volume of the treating group, C_RTV_ is the relative tumor volume of the negative control group.

### mRNA transcriptome sequencing

HCT116 cells were treated with CUS (125, 250 and 500 µM/l), LH (25 µg/ml), DDP (0.5 µg/ml), CUS + DDP and CUS + LH for 48 h. 5–8F cells were treated with CUS (125, 250 µM/l) for 48 h. After washing twice with cold PBS, total RNA was rapidly extracted from the cells with Trizol and samples stored at – 80 °C. Samples were shipped to Guangzhou Epigenic Biology Science and technology LTD for mRNA transcriptome sequencing after qualification of sample RNA. Gene expression analyses were performed using GO (Gene Ontology) and KEGG (Kyoto Encyclopedia of Genes and Genomes) by experts. Genes were filtered using a transcriptome fold change (FC) value of logFC ≥  ± 1.00 and *P* < 0.05. The biological process, molecular function, cell component, signal pathway, and KEGG pathway that were modulated/enriched in cells were analyzed.

### Quantitative reverse transcription PCR (RT-qPCR)

The mRNA transcriptome sequencing data were validated by profiling the expression of 10 differential genes through quantitative reverse transcription PCR (RT-qPCR). Each data point presented for the quantitative PCR assay was derived from three biological replicates (BR) of HCT116 cells treated with CUS, LH, DDP, CUS + LH and CUS + DDP as showing in mRNA Transcriptome Sequencing for 48 h. Total RNA from each BR was extracted with Trizol, reversed transcribed and the cDNA used as a template for qPCR. Primers for two upregulated genes (HMOX1, DDIT3) and eight downregulated genes (MUC1, NCOA5, GJB4, HS3ST1, LFNG, KRT23, CASC19 and SPNS3) were designed according to the relevant target gene sequences published by GenBank (Table 1) and synthesized by Guangzhou Epigenic Biology Science and technology LTD. Quantitative PCR was carried out in a fluorescence quantitative PCR instrument. The thermocycling conditions were 95 °C for 30 s, followed by 40 cycles of 95 °C for 10 s and 60 °C for 30 s. GAPDH was selected as the internal control gene to normalize the gene expression data. Relative quantification of the target gene was calculated by the comparative 2^–ΔΔCT^ method.

**Table 1:** Primer sequences used for RT-qPCR assays.

Gene name	Forward	Reverse
HMOX1	AACTTTCAGAAGGGCCAGGT	GTAGACAGGGGCGAAGACTG
DDIT3	CCCCATTATCCTGCAGATGT	GTCCTCATACCAGGCTTCCA
MUCI	CTCCTTTCTTCCTGCTGCTG	CTCCACCTGGGGTAGAGCTT
NCOA5	ATGAATACGGCTCCATCAAGACC	ACTTCCTCGAATTGGGGATCG
SPNS3	CTGTCTTCGTTAGCTGCCTG	GCTCCTGACCACAGCAAGATAC
CASC19	CAGGGTTCTGGTCATCCCAC	GGTTCTAACCCAGGCACTCC
GJB4	CTGCTGAGTGGCGTGAACAA	CACACGAAAGATGAACACCACA
KRT23	TACTAGGCGGAAATGGGAAGG	TCTTACCATCCACTATCTGCTCC
LFNG	GTCAGCGAGAACAAGGTGC	GATCCGCTCAGCCGTATTCAT
HS3ST1	TGGGAGGAGCATTACAGCCA	ACTTTGGGCGACGTGAAATAC

### Statistical analyses

The data from three or more independent groups were analyzed by one-way ANOVA. Comparison of two groups was carried out by an independent *t*-test. Data are represented as mean ± SD in duplicate assays and analyzed using SPSS statistical software version 21.0. */^#^ indicate *P* < 0.05, **/^##^ indicate *P* < 0.01, and ***/^###^ indicate *P* < 0.001. The acceptable level for statistical significance was *P* < 0.05.

## Results

### Different cephalosporin antibiotics show greatly different anticancer activity on different histological nasopharyngeal carcinoma cells

**Fig. 1:**
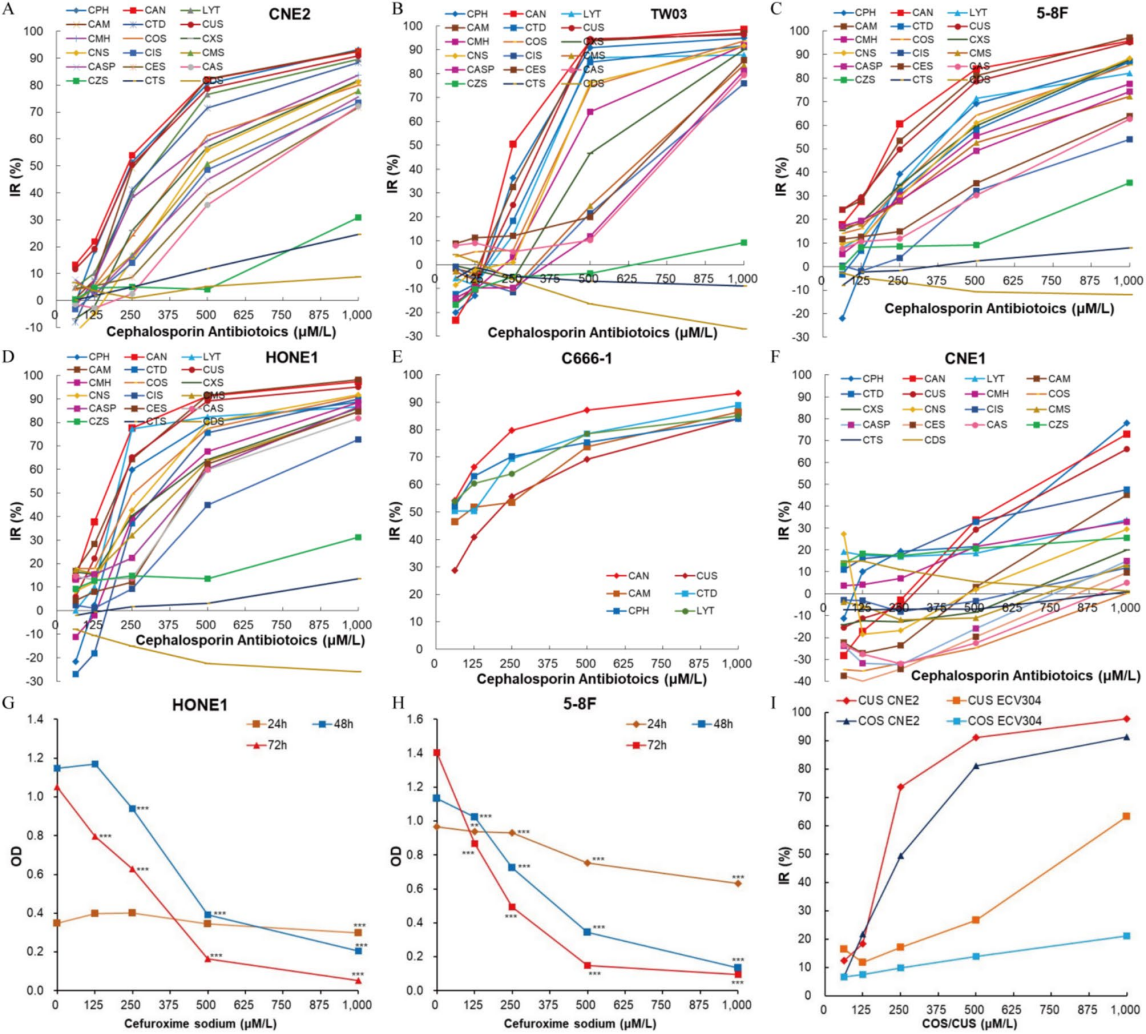
Inhibition of cephalosporin antibiotics on viability of nasopharyngeal cancer cells varies greatly. (**A**) IR of cell viability in CNE2 cells. (**B**) IR of cell viability in TW03 cells. (**C**) IR of cell viability in 5–8F cells. (**D**) IR of cell viability in HONE1 cells. (**E**) IR of cell viability in C666-1 cells. (**F**) IR of cell viability in CNE1 cells. (**G**) time-course of OD in HONE1 cells treated with CUS. (**H**) Time-course of OD in 5–8F cells treated with CUS. (**I**) IR comparation between CNE2 cells and ECV304 cells treated by CUS or COS^10^.

The in vitro anticancer activity of 18 cephalosporin antibiotics (CAM, CUS, CAN, COS, CPH, CMS, LYT, CTD, CMH, CXS, CNS, CIS, CES, CAS, CASP, CZS, CTS and CDS) was evaluated on 6 human nasopharyngeal carcinoma cell lines with different histological characters (CNE1, CNE2, 5-8F, TW03, HONE1, and C666-1). We measured the viability of cancer cells after treatment with cephalosporin antibiotics for 72 h (or 24, 48 and 72 h in the time-course study) at the same time by MTT assay. Results showed that different cephalosporin antibiotics showed greatly different anticancer activity on different nasopharyngeal cancer cell lines (Fig. 1). 15 cephalosporin antibiotics (CAM, CUS, CAN, COS, CPH, CMS, LYT, CTD, CMH, CXS, CNS, CIS, CES, CAS, and CASP) excellently inhibited the viability of nasopharyngeal carcinoma cells except CNE1 in a concentration-dependent manner, while 3 cephalosporin antibiotics (CZS, CTS and CDS) did not show evident anticancer effects (Fig. 1A–F). The well-differentiated CNE1 was relative much less insensitive to all cephalosporin antibiotics compared with other 5 nasopharyngeal cancer cell lines (such as the IC50 of CUS in CNE1 was 755.22 μM/l, much higher than 171.23, 185.36, 197.74, 314.94 and 325.48 μM/l in HONE1, C666-1, 5-8F, TW03 and CNE2 respectively). 9 cephalosporin antibiotics promoted the proliferation of CNE1 cells under this concentration of 500 μM/l (Fig. 1F).

We further investigated the time-dependent nature of CUS inhibition on HONE1 and 5–8F growth. Cell viability decreased a little in HONE1 or slowly in 5–8F after incubation with series concentrations of CUS for 24 h, but then drastically decreased at 48 h or 72 h (Fig. 1G,H). The results suggested the anticancer activity of cephalosporin antibiotics was temporally delayed, which are consistent with previous finding in CNE2. At 1000 μM/l, the optical density (OD) values in 5–8F at 48 h and 72 h or in HONE1 at 72 h were significantly less than the original OD value before CUS administration, which means that CUS could not only inhibit the proliferation of cancer cells, but also further killed the original cells. In previous study^10^, we have demonstrated that cephalosporin antibiotics inhibit the viability of CNE2 cells to a much greater extent than in ECV304 cells (normal human endothelial vein cell line) (Fig. 1I). Cell viability results indicated that, with little or much less cytotoxic effects on normal human cells, cephalosporin antibiotics can be efficaciously used for treatment of nasopharyngeal carcinomas except CNE1.

### Anticancer activity of cephalosporin antibiotics on nasopharyngeal carcinoma cells is regularly determined by the sidechain groups out of β-lactam

**Fig. 2:**
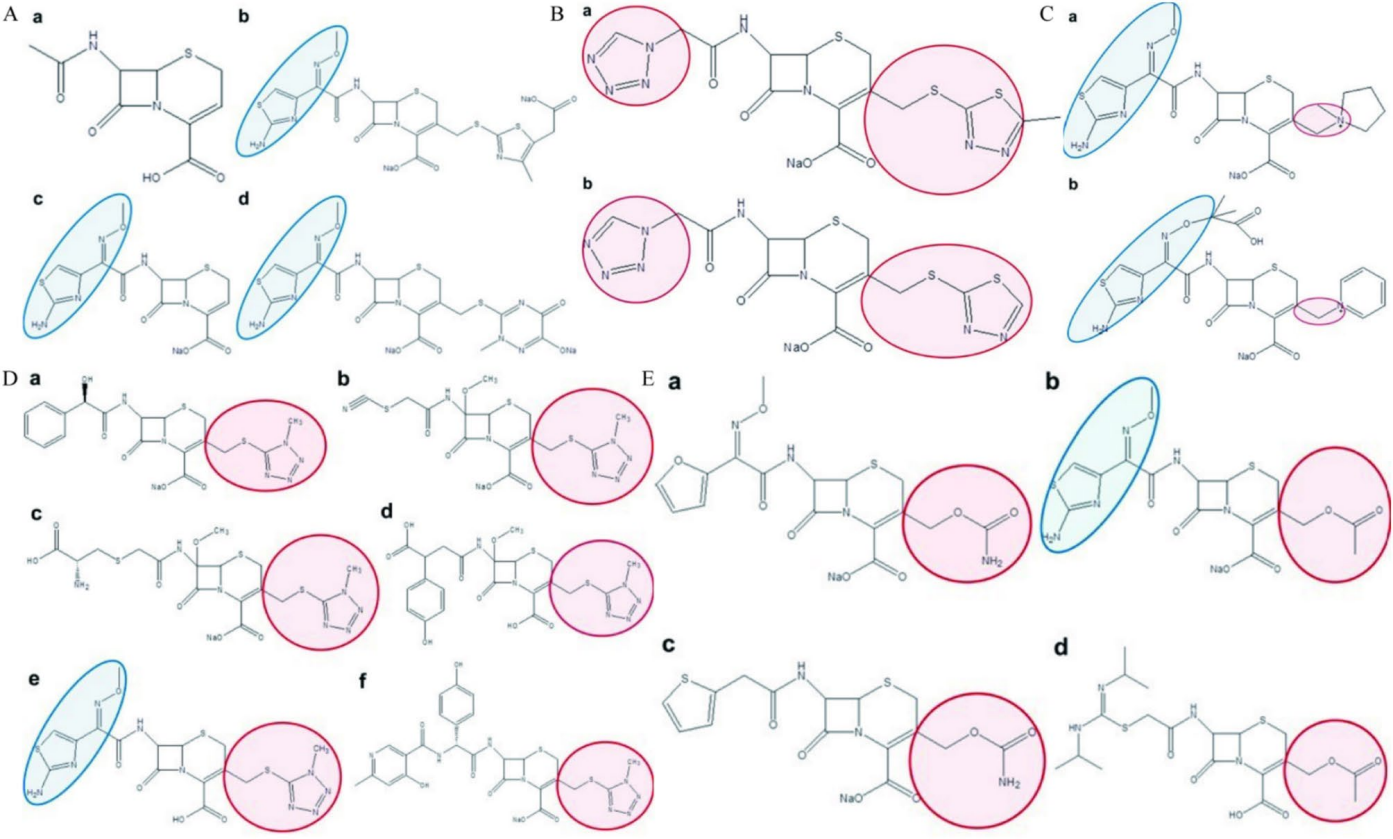
Relationship between chemical structure and anticancer activity on nasopharyngeal carcinoma cells. (**A**) Cephalosporin antibiotics without evident anticancer activity (a, Chemical backbone of cephalosporin antibiotics. b, Cefodizime sodium. c, Ceftizoxime Sodium. d, Ceftriaxone sodium). (**B**) Two anticancer cephalosporin antibiotics which have the same left chemical sidechain group and the same right chemical sidechain group (a, Cefazolin Sodium. b, Ceftezole sodium). (**C**) Two anticancer cephalosporin antibiotics which have the same the same right chemical sidechain group (a, Cefepime hydrochloride. b, Ceftazidime). (**D**) Six anticancer cephalosporin antibiotics which have the same right chemical sidechain group (a, Cefamandole nafate. b, Cefmetazole sodium. c, Cefminox Sodium. d, Layangtoubaona, e, Cefmenoxime hydrochloride. f, Cefpiramide sodium). (**E**) Four anticancer cephalosporin antibiotics which have the same right chemical sidechain group (a, Cefuroxime sodium. b, Cefotaxime sodium. c, Cefoxitin sodium. d, Cefathiamidine).

15 cephalosporin antibiotics showed strong anticancer effects on nasopharyngeal cancer cells in vitro, while ceftriaxone sodium (CTS), cefodizime sodium (CDS) and ceftizoxime sodium (CZS) did not (Fig. 1A–E). All cephalosporin antibiotics are derived from the same chemical backbone 7-(5-amino-5-carboxyvaleramido) cephalosporanic acid (Fig. 2A-a). Therefore, beta-lactam backbone is not directly associated with the anticancer activity of cephalosporin antibiotics.

We further analyzed the relationship between sidechain groups and anticancer activity. Although cefodizime sodium (CDS, Fig. 2A-b), ceftizoxime sodium (CZS, Fig. 2A-c), ceftriaxone sodium (CTS, Fig. 2A-d), and cefepime hydrochloride (CPH, Fig. 2C-a), ceftazidime (CTD, Fig. 2C-b), cefmenoxime hydrochloride (CMH, Fig. 2D-e), cefotaxime sodium (COS, Fig. 2E-b) have the same left sidechain group (circled in blue in Fig. 2), their anticancer activity varied greatly. CDS (Fig. 2A-b), CZS (Fig. 2A-c), and CTS (Fig. 2A-d) had no evident anticancer activity while other antibiotics showed strong anticancer activity on nasopharyngeal cancer cells. We deduce that the left sidechain group circled in blue in Fig. 2 is not the critical group contributing to the anticancer activity. On the other hand, cephalosporin antibiotics which have the same sidechain groups circled in pink in Fig. 2B–E showed strong anticancer activity. Therefore, the 5 sidechain groups circled in pink in Fig. 2 look likely the critical groups supporting the anticancer activity of cephalosporin antibiotics.

### The anticancer activity of cephalosporin antibiotics varies greatly in other types of cancer cells

**Fig. 3:**
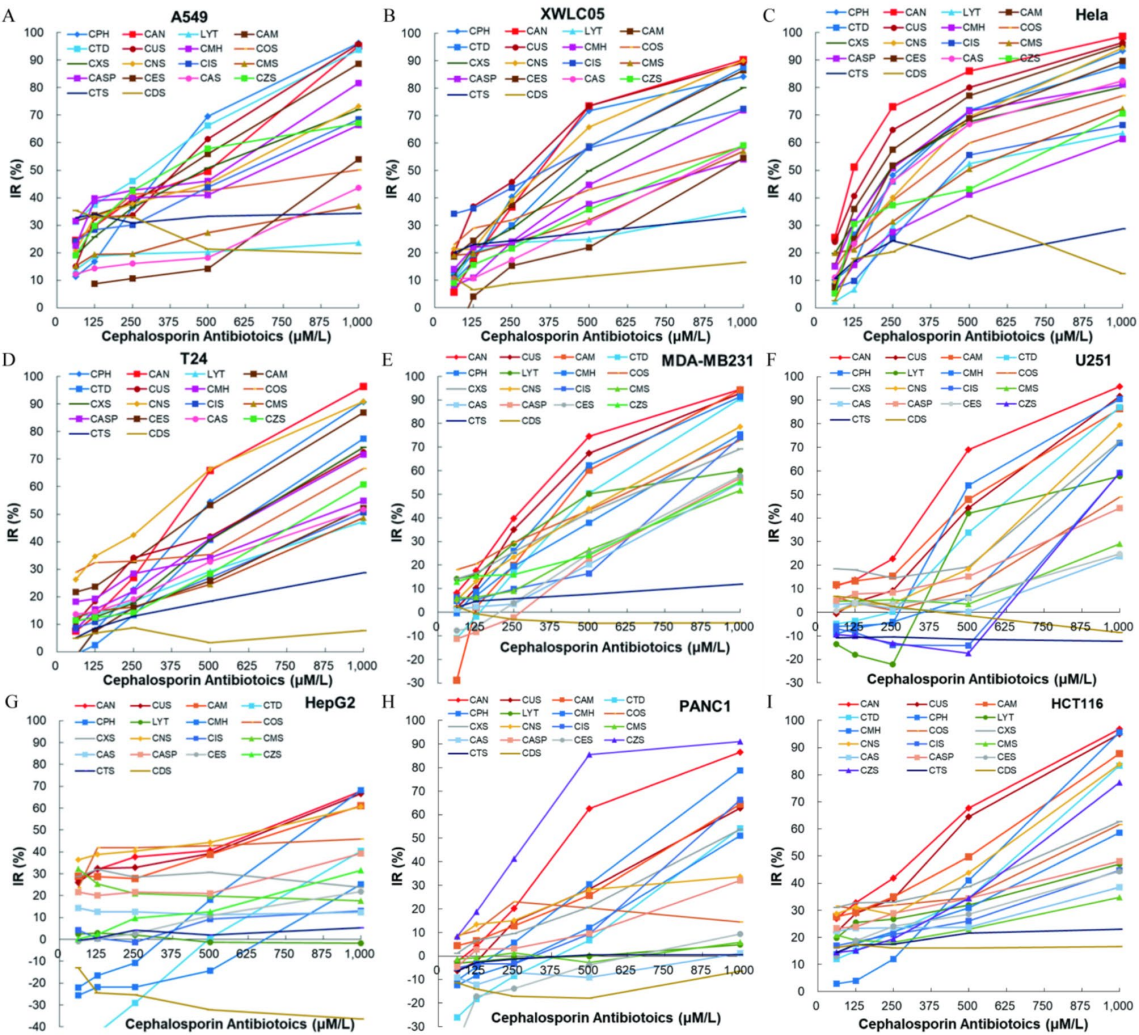
IR of cell viability of 18 cephalosporin antibiotics in other types of cancers except nasopharyngeal cancers. (**A**) A549. (**B**) XWLC05. (**C**) Hela. (**D**) T24. (**E**) MDA-MB231. (**F**) U251. (**G**) HepG2. (**H**) PANC1. (**I**) HCT116.

Inhibition effects of 18 cephalosporin antibiotics on the cell viability of other 10 cancers except nasopharyngeal cancers were also investigated and compared at the same time. With the increasing of antibiotics concentration, except 3 antibiotics (CTS, CDS and CZS), the inhibition effect of other 15 antibiotics on cell viability markedly increased (Fig. 3A–I, Fig. S1A(NCI-H460)). The inhibition tendency in these 10 cancer cell lines were consistent with the results we observed in nasopharyngeal cancer cell lines. The inhibition effects of the 15 anticancer antibiotics on the cell viability of 10 cancers varied greatly (such as the IC50 of CUS in H460, Hela, XWLC05, HCT116, A549, MDA-MB231, U251, T24, PANC1 and HepG2 were 116.45, 157.91, 217.54, 255.79, 269.95, 337.05, 498.61, 499.23, 571.33 and 572.19 μM/l respectively). Interestingly, all cancer cells (including NPC cells) were insensitive to ceftizoxime sodium (CZS) except PANC1. PANC1 (Fig. 3H) were insensitive to most of cephalosporin antibiotics except CZS, CAN, CUS, CPH and CAM (IC50 were 258.42, 430.20, 571.33, 645.85 and 829.37 μM/l respectively).

**Fig. 4:**
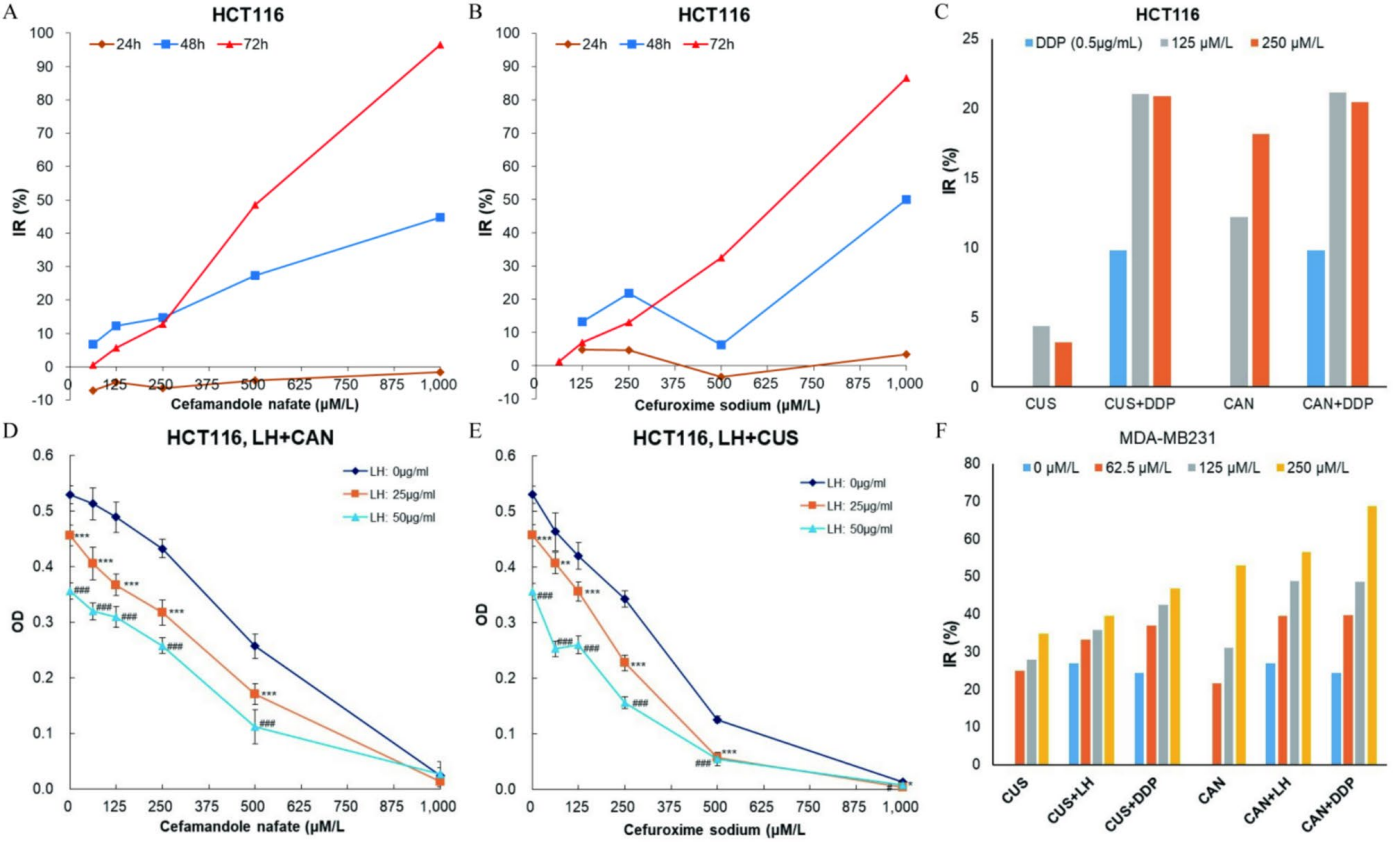
The time course of cell viability inhibition of CAN and CUS, and their combination cytotoxicity with anticancer agents. (**A**) The time course of viability inhibition of CAN on HCT116 cells. (**B**) The time course of viability inhibition of CUS on HCT116 cells. (**C**) The combination cytotoxicity (IR) of CAN + DDP and CUS + DDP on HCT116 cells. (**D**) The combination cytotoxicity (OD) of CAN + LH on HCT116 cells. (**E**) The combination cytotoxicity (OD) of CUS + LH on HCT116 cells. (**F**) The combination cytotoxicity (IR) of CAN + DDP and CUS + DDP on MDA-MB231 cells.

We further investigated the time-dependent nature of CAN and CUS inhibition on HCT116 growth. Results showed that the inhibition of cell viability in HCT116 did not significantly change after incubation with CAN or CUS for 24 h at all concentrations, but drastically increased when treated for 48 or 72 h (Fig. 4A,B). IR increased with the increasing of treating time 24 h later. These results suggested the anticancer activity of cephalosporin antibiotics was temporally delayed. The time course of cell viability inhibition observed in HCT116 was consistent with the results observed in HONE1 cells treated with CUS (Fig. 1G), 5–8F cells treated with CUS (Fig. 1H) and CNE2 cells treated with COS^10^. MTT OD levels in HCT116 cells treated with 500 or 1000 μM/l CAN or CUS returned to or was significantly less than the original OD values before drug administration. Results of the time-course experiments indicated that the anticancer effect of cephalosporin antibiotics was time- and concentration-dependent, but was temporally delayed.

### Cefuroxime significantly inhibits clone formation of cancer cells, and enhances the anti-proliferation efficacy of other anticancer agents

The effects of CUS, CUS + LH and CUS + DDP on the clone formation of HCT116 cells and 5–8F cells were investigated. As expected, as positive anticancer agents, DDP and LH greatly inhibited clone formation of both HCT116 (Fig. S1D,H) and 5-8F(Fig. S1C,E) cells (*p* < 0.05 or *p* < 0.001) . With the increasing of CUS concentration (125, 250 and 500 μM/l), the number of clonogenic colonies that grew for HCT116 (Fig. S1D) and 5–8F (Fig. S1C) gradually reduced. When medium contained 0.5 μg/ml of DDP or 25 μg/ml of LH, the clone formation of HCT116 cells (Fig. S1D) or 5–8F cells (Fig. S1C) treated by CUS at each CUS concentration was further significantly inhibited. Quantitatively, the number of HCT16 and 5–8F clones after CUS treatment were significantly less than those of the negative control group (NC) (*p* < 0.01), and the colony number that grew in combination groups was further significantly decreased (*p* < 0.05, 0.01 or 0.001) and the IR of colony number significantly increased.

### Cefuroxime arrests cell cycle and promotes apoptosis differently in different cancer cells

The cell cycle of HCT116 and 5–8F cells was determined by flow cytometry (FCM) after 48 h of CUS treatment. With increasing CUS concentrations, the proportion of 5–8F cells in G0/G1 phase decreased drastically while the proportions of cells in G2/M and S phases drastically increased (Fig. S1F,K). 5–8F cells treated by CUS progressed into S phase and were arrested in G2/M phase, which are consistent with previous discovery in CNE2 cells treated by COS^10^. However, cell cycle distribution of HCT116 cells treated by CUS was different from that of 5–8F cells, in which, with increasing CUS concentration, the proportion of HCT116 cells in G2/M phase decreased gradually while the proportion of cells in S phase increased (Fig. S1I, S1M). CUS arrested the cell cycle of HCT116 cells at S phase.

FCM was also used to detect the apoptosis induced by CUS. HCT116 and 5–8F cells were treated with series concentrations of CUS for 48 h. Early apoptosis (EA) rate of HCT116 cells increased markedly with increasing CUS concentration (Fig. S1J, S1N, P < 0.001), and late apoptosis (LA) rate changed a little. On the contrary, late apoptosis (LA) induced in 5–8F cells markedly increased with increasing CUS concentration (Fig. S1G, S1L, P < 0.01) but early apoptosis (EA) rate changed a little. The total apoptosis (TA) rates were 1.16%, 11.18%, 17.50% and 18.23% in HCT116 cells; 1.98%, 3.29%, 9.05% and 11.20% in 5-8F cells treating with series concentrations of CUS (0, 100, 200, and 300 μM/l) respectively. Apoptosis induced by CUS was concentration-dependent, which is consistent with the results from cell viability experiment.

### Combination of anticancer cephalosporin antibiotics and other anticancer agents enhances the cytotoxicity in cancer cells

The combination cytotoxicity of CAN or CUS with DDP or LH was evaluated in HCT116 and MDA-MB231 cells. Viability of HCT116 cells was measured after HCT116 cells were intervened with series concentrations of CAN or CUS (0, 62.5, 125 µM/L) in DDP medium (DDP: 0.5 µg/ml) (Fig. 4C), or series concentrations of CAN or CUS (0, 62.5, 125, 250, 500 and 1000 µM/l) in LH medium (LH: 25, 50 µg/ml) (Fig. 4D,E) for 72 h. Viability of MDA-MB231 cells was measured after cancer cells were treated with series concentrations of CAN or CUS (0, 62.5, 125 and 250 µM/l) in DDP medium (DDP: 0.5 µg/ml) or LH medium (LH: 25 µg/ml) (Fig. 4F).

IR (inhibition rate) of viability in HCT116 cells treated by CAN + DDP or CUS + DDP were promoted and but did not increase with the increasing of antibiotics concentration at 125 and 250 µM/L (Fig. 4C). CAN and CUS concentration-dependently reduced the viability of HCT116 cells in LH-free medium (Fig. 4D,E; LH: 0 µg/ml) as we found in Fig. 3I. When the medium containing 25 or 50 µg/ml of LH, OD in CAN + LH group and CUS + LH group was significantly reduced (Fig. 4D,E; LH: 25 or 50 µg/ml) (*p* < 0.01 or 0.001). The same results were observed in MDA-MB231 cells that CAN and CUS concentration-dependently enhanced the cytotoxicity of LH or DDP when they were concomitantly used (Fig. 4F).

Q values assessed by the evaluation of drug combination index indicated that combination of CAN (Table 2) or CUS (Table 3) with LH or DDP showed additive or synergistic anticancer effect. CAN and CUS can enhanced the cytotoxicity of DDP or LH on cancer cells when they were concomitantly used. Ceftriaxone sodium (CTS), which has no evident anticancer activity (Figs. 1, 2,^10^), also could not evidently promote the cytotoxicity of levofloxacin (Fig. S1B).

**Table 2:** Q values of CAN in different cancer cell lines by the evaluation of drug combination index.

CUS (µM/l)	HCT116			MDA-MB231	
	LH (25 µg/ml)	LH (50 µg/ml)	DDP (0.5 µg/ml)	LH (25 µg/ml)	DDP (0.5 µg/ml)
62.5	1.4152	1.1342			
125	1.4992	1.0928	1.0145	0.7365	0.8518
250	1.3485	1.1363	0.7792	0.7578	0.9335
500	1.1651	1.1682		0.7522	0.9230
1000	1.0138	0.9787			

Antagonistic effect: Q ≤ 0.85. Additive effect: 0.85 < Q < 1.15. Synergistic effect: Q ≥ 1.15.

**Table 3:** Q values of CUS in different cancer cell lines by the evaluation of drug combination index.

CAN (µM/l)	HCT116		MDA-MB231	
	LH (25 µg/ml)	DDP (0.5 µg/ml)	LH (25 µg/ml)	DDP (0.5 µg/ml)
62.5	0.9391			
125	1.0323	1.5280	0.9257	0.9765
250	1.2877	1.6436	0.9844	1.0142
500	1.1228		0.8607	1.0668
1000	1.0132			

Antagonistic effect: Q ≤ 0.85. Additive effect: 0.85 < Q < 1.15. Synergistic effect: Q ≥ 1.15.

### Cefuroxime significantly inhibits the growth of colorectal carcinoma xenograft in nude mice, and combination of cefuroxime and cisplatin further suppresses tumor growth

**Fig. 5:**
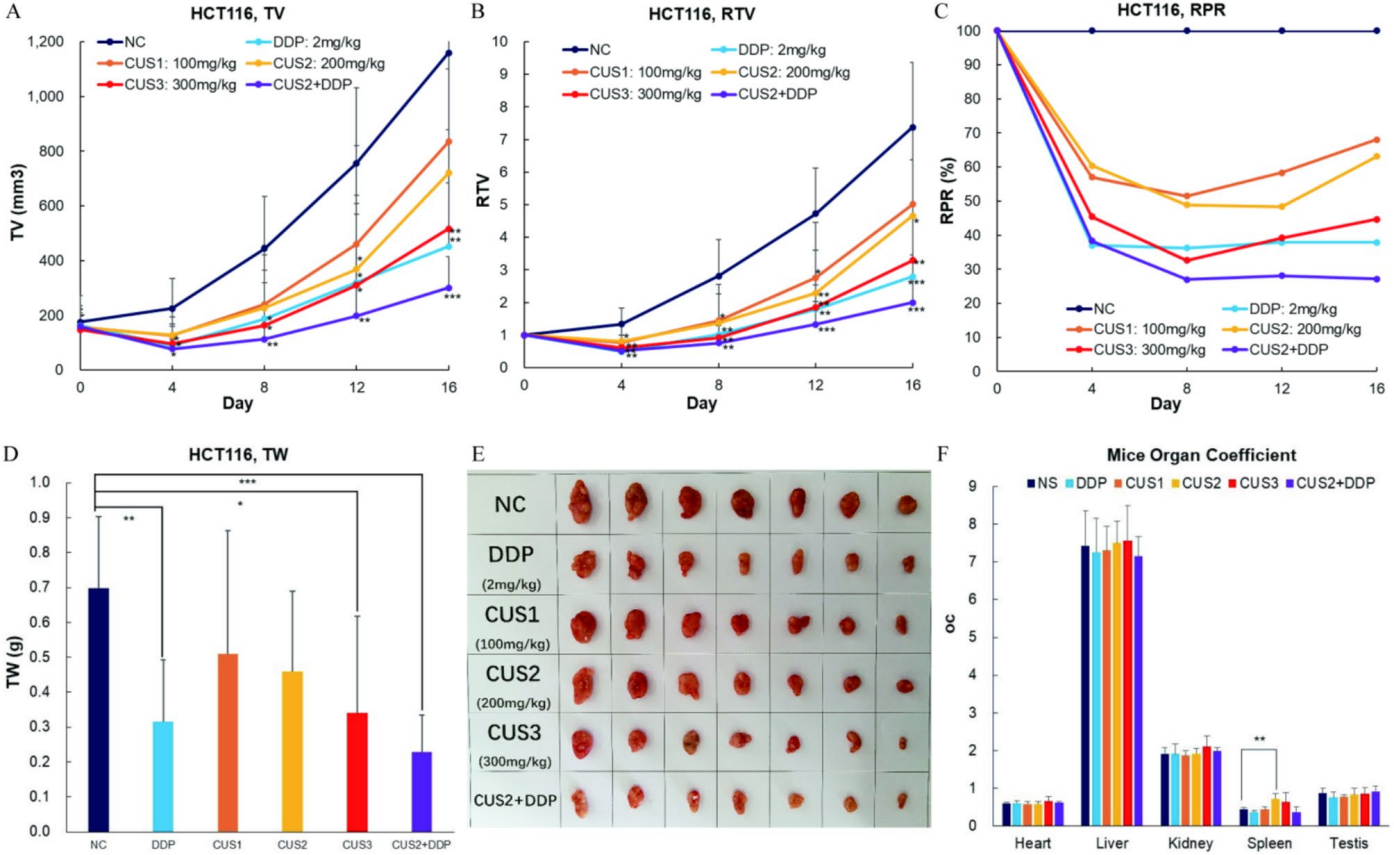
Effects of cefuroxime, cisplatin and their combination on the HCT116 tumor growth in xenograft mouse model. (**A**) TV throughout the study in different groups. (**B**) RTV throughout the study in different groups. (**C**) RPR throughout the study in different groups. (**D**) TW at the end of the study in different groups. (**E**) The ex vivo tumor photos in different groups. (**F**) OC (organ coefficient) at the end of the study in different groups. Data are mean ± SD from 7 mice. Compared with NC group: *P < 0.05, **P < 0.01, ***P < 0.001.

Next, the anticancer activity of CUS and CUS + DDP in vivo were evaluated in a HCT116 xenograft model using balb/c nude mice. The average original tumor volumes (TV0) in each group before drug administration had no statistically difference (Fig. 5A) (*p* > 0.05). Mice were randomly divided into 6 group: negative control group (NC group, neutral saline), positive control group (DDP group, 2 mg/kg.bw), 3 CUS groups (100, 200, and 300 mg/kg.bw in CUS1, CUS2 and CUS3 group respectively), and CUS2 + DDP group.

As a positive anticancer drug, DDP showed significant anticancer effects on HCT116 xenografts (Fig. 5A–E), in which TV (tumor volume), RTV (relative tumor volume) and TW (tumor weight) in DDP group were significantly less than those in NC group (*p* < 0.05 or 0.01). CUS effectively inhibited the growth of HCT116 tumors, and this growth inhibition effect increased with the increasing of CUS doses (Fig. 5A–E). During the period of drug treatment, the TV and the RTV in CUS2 group and CUS3 group from day 4 on were significantly less than those measured in NC group (Fig. 5A) (*p* < 0.05, or 0.01). At the end of the experiment, the relative tumor volumes (RTV4) in response to 100, 200, and 300 mg/kg CUS were 5.02-, 4.66- (*p* < 0.05), and 3.29- (*p* < 0.01) fold of the original tumor volumes (TV0) respectively, and markedly less than that of NC group (7.38-fold) (Fig. 4B). Although TV (Fig. 5A) and RTV (Fig. 5B) in the CUS treating groups increased with time, tumor growth at all measure times was significantly inhibited by CUS in a dose-dependent manner. The RPR at day 8 (RPR2) in CUS treating groups was 51.48%, 48.92% and 32.64% respectively (the lowest in the whole experiment) relative to the NC group. The RPR at day 12 and day 16 in the CUS treating groups were higher than those measured at day 8, which might be resulted from the degradation of CUS (CUS was prepared at day 0). The inhibition effect of CUS on the growth of HCT116 tumor can also be seen by the average ex vivo tumor weight (TW) at the end of the experiment (Fig. 5D). The average tumor weights in CUS groups were 0.51 g, 0.46 g, and 0.34 g respectively, which were also markedly less than 0.70 g in the NC group (*p* < 0.05 in CUS3 group).

Tumor volumes in CUS2 + DDP group were 77.00, 113.10, 197.68 and 300.32 mm^3^ at day 4, 8, 12 and 16, significantly less than 224.60, 443.57, 755.16 and 1159.24 mm^3^ in NC group respectively (*p* < 0.05, 0.01, 0.01 and 0.001), and much less than 88.95, 187.10, 320.23 and 451.08 mm^3^ in DDP group (*p* > 0.05) and 128.11, 227.35, 367.94 and 720.34 mm^3^ in CUS2 group (*p* > 0.05) (Fig. 5A). RTV were 0.51-, 0.76-, 1.33- and 2.00-fold of TV0 at day 4, 8, 12 and 16 in CUS2 + DDP group, significantly less than 1.34-, 2.81-, 4.72- and 7.38-fold in NC group (*p* < 0.01, 0.01, 0.001 and 0.001 respectively), and much less than 0.49-, 1.0-, 1.79- and 2.80-fold in DDP group and 0.81-, 1.38-, 2.28- and 4.66-fold in CUS2 group respectively (Fig. 4B). RPR in CUS2 + DDP group were 38.34%, 26.95%, 28.11% and 27.10% at day 4, 8, 12 and 16, relative to the NC group. RPR in CUS2 + DDP group decreased with treating time, and were much less than those in NC group, CUS2 group, or DDP group throughout the study (Fig. 5C). Compared with NC group, TV in CUS2 + DDP group increased slowly since drug treatment. Tumor growth inhibition of CUS2 + DDP was better than that of CUS2 or DDP alone (Fig. 5A–E). At the end of the experiment, TW in CUS2 + DDP group was 0.23 g, significantly less than 0.70 g in NC group (*p* < 0.001), and much less than 0.32 g in DDP group and 0.46 g in CUS2 group (Fig. 5D). The anticancer efficacy and chemosensitizing efficacy of cefuroxime can also be judged by the ex vivo tumor size (Fig. 5E). CUS, DDP and CUS + DDP effectively inhibited HCT116 tumor growth, and the anticancer efficacy of CUS + DDP was much better than that of CUS or DDP alone.

Compared with NC group, the development of heart, liver, kidney, testis and spleen of mice in CUS groups, DDP group and in CUS2 + DDP group were not statistically different from that in NC group except the spleen coefficient in CUS2 group was higher (Fig. 5F) (*p* < 0.01). The results indicated that cefuroxime was a safe anticancer agent and chemosensitizer against HCT116 cancer in vivo.

### Cefuroxime and cefotaxime regulate genes in common in direction favoring the anticancer activity in different cancer cells, and combination of cefuroxime and cisplatin or levofloxacin regulates anticancer genes in common supporting the enhancement of anticancer efficacy

To reveal the possible common molecular mechanisms by which CUS becomes cytotoxic to different cancer cells and why combination of CUS and DDP or LH enhances anticancer efficacy, we investigated potential changes in gene expression in different cancer cells (HCT116 and 5–8F). RNA was isolated from HCT116 cells treated with CUS (125, 250 and 500 µM/l), CUS + DDP (125, 250 and 500 µM/l CUS in 0.5 μg/ml DDP medium respectively) and CUS + LH (125, 250 and 500 µM/l CUS in 25 μg/ml LH medium respectively) for 48 h, or isolated from 5-8F cells treated with CUS (125 and 250 µM/l) for 48 h. RNA was analyzed by mRNA transcriptome sequencing and GO (Gene Ontology) enrichment analysis. Present results are further compared with previous results found in CNE2 cells treated by COS^10^.

**Fig. 6:**
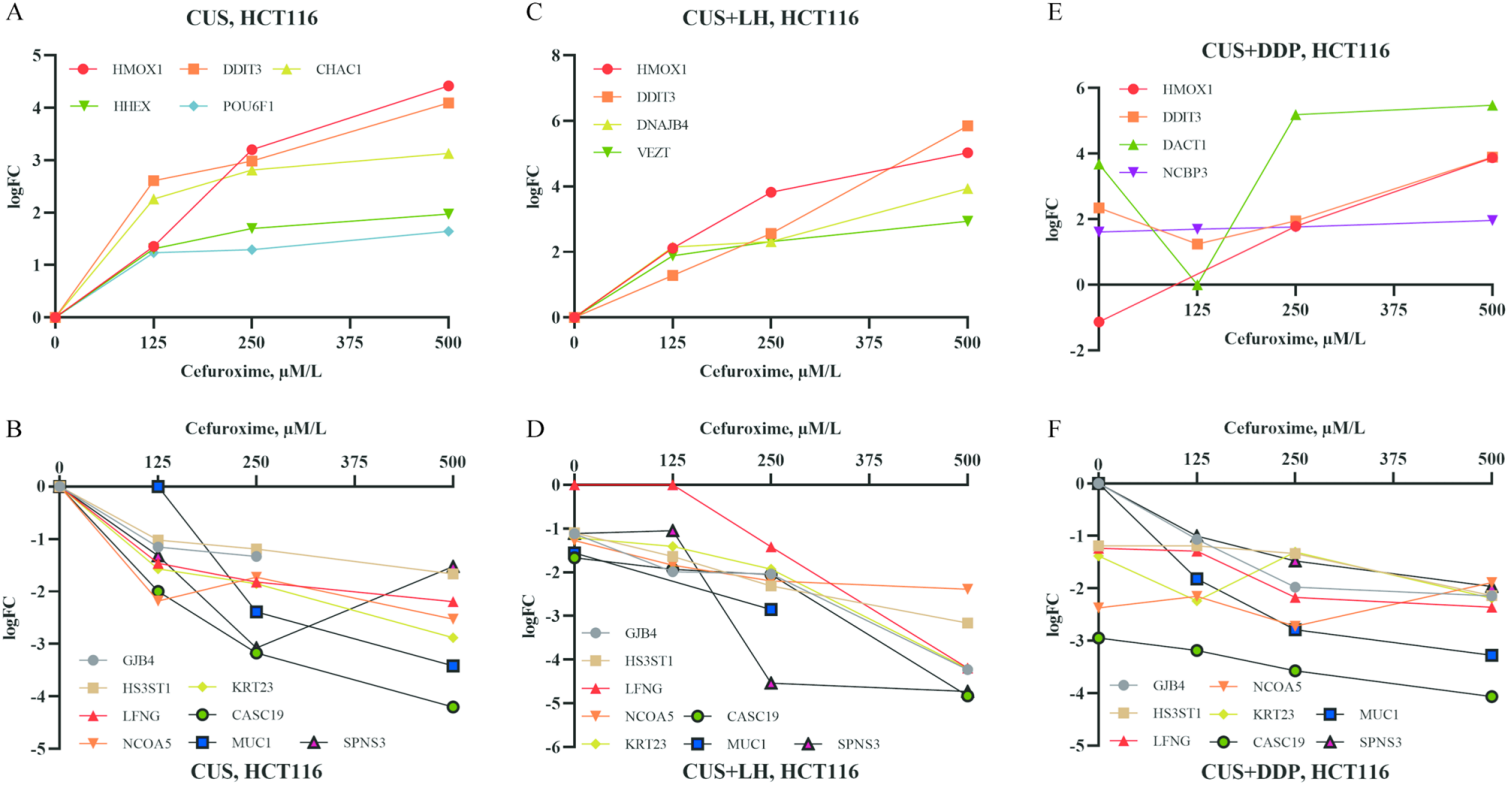
Cefuroxime, combination of cefuroxime and anticancer agents regulate genes in a direction that favors anticancer activity. (**A**) The differential anticancer genes upregulated by CUS. (**B**) The differential anticancer genes downregulated by CUS. (**C**) The differential anticancer genes upregulated by CUS + LH. (**D**) The differential anticancer genes downregulated by CUS + LH. (**E**) The differential anticancer genes upregulated by CUS + DDP. (**F**) The differential anticancer genes downregulated by CUS + DDP.

2220(Fig. S2A, CUS125_NC_DEGs_2220.txt), 2323(Fig. S2B, CUS250_NC_DEGs_2323.txt) and 2411(Fig. S2C, CUS500_NC_DEGs_2411) transcripts were noted to be differentially regulated in HCT116 cells at CUS concentration of 125, 250, and 500 µM/L using a cut-off logFC (fold change) ≥  ± 1.00 and p < 0.05. Among these differential genes, HMOX1, DDIT3, CHAC1, HHEX, and POU6F1 were upregulated (Fig. 6A) and MUC1, NCOA5, HS3ST1, LFNG, KRT23, CASC19, GJB4, and SPNS3 were downregulated (Fig. 6B) by CUS in a concentration-dependent manner. Regulations of these 13 genes in the direction are reported to favor anticancer activity and therefore these genes likely contributed to the anticancer activity of CUS on HCT116 cells.

2053(Fig. S2J, DDP_DEGs_2053.txt), 2449(Fig. S2D, CUS125_DDP_DEGs_2449.txt), 2528(Fig. S2E, CUS250_DDP_DEGs_2528.txt) and 2877(Fig. S2F, CUS500_DDP_DEGs_2877.txt) transcripts were noted to be differentially regulated at CUS concentration of 0, 125, 250, and 500 µM/l in DDP medium (DDP: 0.5 µg/ml) (CUS + DDP group) using a cut-off logFC (fold change) ≥  ± 1.00 and p < 0.05. Among these differential genes, HMOX1, DDIT3, DACT1, and NCBP3 were upregulated (Fig. 6E) and the 8 downregulated genes regulated by CUS alone were also downregulated (Fig. 6F) by CUS + DDP in a CUS-concentration dependent manner. Regulations of these 12 genes in the direction are reported to favor anticancer activity and therefore they likely contributed to the anticancer efficacy of CUS + DDP on HCT116 cells. Furthermore, compared with CUS groups and DDP group, the logFC values of 5 down-genes (GJB4, LFNG, HS3ST1, CASC19 and MUC1) were further reduced in the CUS + DDP groups, which means these 5 down-genes likely contributed to the enhancement of anticancer efficacy in CUS + DDP groups.

2315(Fig. S2K, LH_DEGs_2315.txt), 2542(Fig. S2G, CUS125_LH25_DEGs_2542.txt), 2966(Fig. S2H, CUS250_LH25_DEGs_2966.txt) and 5438(Fig. S2I, CUS500_LH25_DEGs_5438.txt) transcripts were noted to be differentially regulated at CUS concentration of 0, 125, 250, and 500 µM/l in LH medium (LH: 25 µg/ml) (CUS + LH group) using a cut-off logFC (fold change) ≥  ± 1.00 and p < 0.05. Among these differential genes, HMOX1, DDIT3, DNAJB4, and VEZT were upregulated (Fig. 6C), and the 8 downregulated genes regulated by CUS alone were also downregulated (Fig. 6D) by CUS + LH in a CUS-concentration dependent manner. Regulations of these 12 genes in the direction are reported to favor anticancer activity and therefore these genes likely contributed to the anticancer efficacy of CUS + LH on HCT116 cells. Furthermore, compared with CUS groups and LH group, the logFC values of two up-genes (HMOX1 and DDIT3) were further enhanced and 7 down-genes (SPNS3, NCOA5, HS3ST1, LFNG, KRT23, CASC19, and GJB4) were further reduced in the CUS + LH groups, which means these 9 genes likely contributed to the enhancement of anticancer efficacy in CUS + LH groups.

Furthermore, intrinsic apoptotic signaling pathway is the only common apoptotic pathway in which both HMOX1 and DDIT3 overlapped in COS groups^10^, CUS groups, CUS + LH groups and CUS + DDP groups. Intrinsic apoptotic signaling pathway might be the critical chemotherapeutic target of cephalosporin antibiotics.

**Fig. 7:**
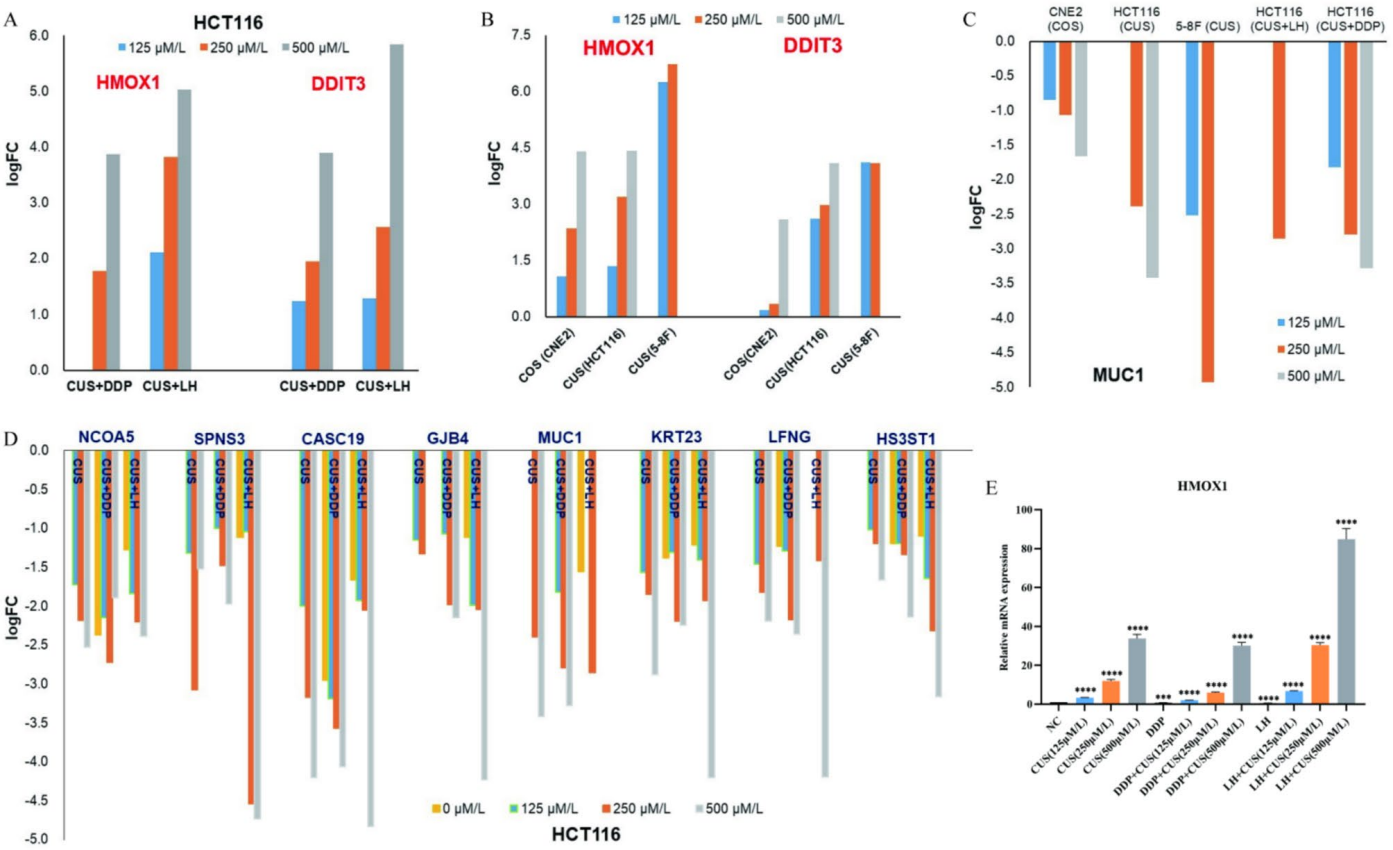
Analysis of the common cancer-associated genes. (**A**) HMOX1 and DDIT3 are the two up-genes commonly regulated by different cephalosporin antibiotics in different cancers in an antibiotic-concentration dependent manner. (**B**) HMOX1 and DDIT3 are the two up-genes commonly regulated by the combination of CUS and DDP or LH in HCT116 cells in a CUS-concentration dependent manner. (**C**) MUC1 is the down-gene commonly regulated by different cephalosporin antibiotics in different cancer cells in an antibiotic-concentration dependent manner. (**D**) 8 down-genes regulated in HC116 cells in a CUS-concentration dependent manner no matter CUS was used independently or concomitantly with DDP or LH. E. HMOX1 mRNA expression in different combination-groups.

The differential anticancer genes regulated by CUS in HCT116 cells are further compared with the genes regulated by CUS in 5–8F cells, and the genes regulated by COS in CNE2 cells^10^. Very interestingly, HMOX1 and DDIT3 were commonly up-regulated (Fig. 7A,B) and MUC1 was commonly down-regulated (Fig. 7C) by COS in CNE2 cells^10^, by CUS in HCT116 cells, by CUS in 5–8F cells, by CUS + LH in HCT116 cells, and by CUS + DDP in HCT116 cells in an antibiotic-concentration dependent manner. Therefore, HMOX1, DDIT3 and MUC1 look likely the common chemotherapeutic targets of different cephalosporin antibiotics in the same cancer cell line or the same cephalosporin antibiotic in different cancer cell lines, no matter these cephalosporin antibiotics are used independently or concomitantly with other anticancer agents. Furthermore, combined with the analysis of apoptotic pathways in CUS groups, CUS + LH groups and CUS + DDP groups, it could be deduced that HMOX1 was the unique upgene contributed to apoptosis induction of anticancer cephalosporin antibiotics.

In addition to MUC1, other 7 down-genes (NCOA5, HS3ST1, LFNG, KRT23, GJB4, CASC19 and SPNS3) were also concentration-dependently downregulated by CUS no matter CUS was used independently or concomitantly with DDP or LH (Fig. 7D). Therefore, downregulation of NCOA5, HS3ST1, LFNG, KRT23, GJB4, CASC19 and SPNS3 were also the common chemotherapeutic targets no matter CUS was used independently or concomitantly with other anticancer agents. Compared with the logFC levels in CUS group, LH group and DDP group, the logFC level of GJB4, LFNG, HS3ST1 and CASC19 were further reduced in CUS + DDP group and in CUS + LH group in a CUS-concentration dependent manner (Fig. 7D). Therefore, co-regulation of these 4 down-genes (GJB4, LFNG, HS3ST1 and CASC19) contributed to the enhancement of anticancer efficacy in the combination of CUS and other anticancer agents.

However, these 7 downregulated genes (HS3ST1, LFNG, KRT23, GJB4, CASC19, NCOA5 and SPNS3) and 3 upregulated genes (CHAC1, HHEX and POU6F1) were not differentially regulated by COS in CNE2 cells or by CUS in 5–8F cells, which means that, except 3 common regulated genes (HMOX1, DDIT3 and MUC1), each anticancer cephalosporin antibiotic has its own unique targeted genes in different cancers. These findings give a scientific explanation why the same cephalosporin antibiotic shows different anticancer efficacy in different cancers, and why different cephalosporin antibiotics show greatly different anticancer efficacy in the same cancer.

We used RT-qPCR to validate the expression level of the 10 differentially expressed genes (HMOX1, DDIT3, MUC1, HS3ST1, LFNG, KRT23, GJB4, CASC19, NCOA5 and SPNS3) identified by mRNA transcriptome sequencing analysis in Fig. 7E and Supplementary Fig. 3A-I in CUS groups, CUS + LH groups and CUS + DDP groups. These genes were selected based on their important roles in anticancer processes as well as their concentration-dependent sensitivity to CUS in HCT116 cells in mRNA transcriptome sequencing data. Their differential expression likely made a significant contribution to the anticancer activity of CUS, and the promotion of anticancer efficacy in CUS + LH and CUS + DDP. Notably, compared to the control group, the mRNA expression of HMOX1 was upregulated by nearly 40-fold after treatment with CUS alone and reached up to 80-fold in the combination therapy (CUS + LH). This suggests that HMOX1 is likely a specific target through which CUS exerts its anticancer effects.

## Discussion

Drug repositioning—the strategic redirection of approved therapeutics to novel disease targets—has emerged as a cost-efficient paradigm for anticancer discovery, particularly when leveraging agents with established safety profiles like cephalosporins^13,14^. This approach gains particular relevance given the emerging understanding that chronic inflammation—recognized as a cancer hallmark—creates a pathological microenvironment conducive to carcinogenesis^15^. Notably, cephalosporins, a major class of β-lactam antibiotics with established safety profiles in infectious disease management, demonstrate unexpected potential in this context. Our findings extend previous observations of antibiotic-mediated tumor suppression^16^, specifically identifying cephalosporins as promising candidates for nasopharyngeal carcinoma (NPC) treatment and chemosensitization^10,12^.

Structural Determinants of Anticancer Activity Through comprehensive structure–activity analysis, we establish that anticancer efficacy in cephalosporins correlates with specific sidechain modifications rather than the conserved β-lactam core or β-lactamase inhibition capacity^17^. This conclusion is supported by three key observations: First, among 15 cephalosporins demonstrating potent in vitro activity against NPC cells, structural analogs ceftriaxone, ceftizoxime, and cefodizime showed no antitumor effects. Second, penicillin derivatives—despite sharing the β-lactam backbone—exhibited negligible activity across tested malignancies. Third, systematic comparison of active versus inactive cephalosporins revealed conserved structure–function patterns in their variable sidechain regions.

Therapeutic Implications Two cephalosporins emerged with particular clinical relevance: Cefamandole nafate (CAN) and cefuroxime sodium (CUS) displayed marked efficacy against both NPC and colorectal cancer, with additionally enhancing cisplatin chemosensitivity. These findings align with our prior identification of levofloxacin (LH) as a broad-spectrum anticancer agent^11^, suggesting that antibiotic repurposing may address dual clinical needs—combating malignancies while managing infection risks in immunocompromised patients. Notably, we identified synergistic anticancer effects between CUS and LH, indicating potential combination therapy applications.

Mechanistic Considerations While the complete anticancer mechanism requires further elucidation, our data suggest conserved molecular targets across different cephalosporins and cancer types. This target conservation persists whether agents are administered as monotherapy or in combination regimens. The structural rules governing cephalosporin activity, as delineated in this study, offer a rational framework for designing optimized β-lactam derivatives with enhanced anticancer properties.

Our prior identification of cephalosporins as tumor-selective agents in nasopharyngeal carcinoma^10^ prompted investigation into their broader applicability to inflammation-associated malignancies, particularly colorectal cancer (CRC). CRC pathogenesis is inextricably linked to chronic inflammatory insults—whether from genetic predisposition, environmental carcinogens, or sustained mucosal inflammation^15^. This connection is exemplified by inflammatory bowel disease (IBD) patients, who exhibit two- to three-fold elevated CRC risk proportional to inflammatory burden^18^, where unresolved inflammation drives carcinogenesis through three synergistic axes: (1) ROS-mediated DNA damage and genomic instability, (2) protumorigenic cytokine signaling, and (3) microbiome-driven tumor microenvironment remodeling^19^. These mechanisms coalesce into an "inflammation-carcinogenesis transition" (ICT)^20–22^—a therapeutic vulnerability our work exploits through cephalosporins’ dual anti-inflammatory/antitumor properties.

Building on cephalosporins’ established perioperative use in CRC^16^, we demonstrate that cefuroxime (CUS) exerts dose-dependent tumor suppression in HCT116 xenografts without hematological or hepatic toxicity. This efficacy stems from CUS’s unique capacity to simultaneously target CRC’s inflammatory drivers and iron-redox vulnerabilities. Transcriptomic profiling revealed CUS induces unprecedented HMOX1 upregulation—nearly 40-fold in monotherapy, 30-fold with DDP coadministration and 80-fold with LH coadministration—directly coupling β-lactam exposure to heme oxygenase-1 overexpression. As the rate-limiting enzyme in ferroptosis, HMOX1 tilts cellular iron homeostasis toward catastrophe that eliminate malignancy-permissive stromal niches^23,24^. The HMOX1-ferroptosis axis thus emerges as both mechanistic linchpin and therapeutic biomarker. These findings not only establish a molecular framework for CUS-mediated CRC inhibition but also provide a rationale for leveraging HMOX1 as a predictive biomarker in future clinical.

The ferroptosis regulator HMOX1—a ubiquitously expressed heme catabolism enzyme—serves as the molecular linchpin connecting cephalosporins’ antimicrobial and antitumor actions. By catalyzing heme degradation into Fe^2+^, biliverdin, and CO, HMOX1 governs cellular iron flux and redox equilibrium^23,24^. Our work establishes that HMOX1 overexpression (nearly 40-fold induction by CUS monotherapy; 30-fold with DDP; 80-fold with LH) licenses ferroptosis through two convergent pathways: (1) Fe^2+^ overload via TFR1 upregulation and FTH1/FTL suppression, and (2) ROS amplification via Fenton-driven lipid peroxidation (PLOOH/MDA accumulation)^25–34^. These effects culminate in mitochondrial catastrophe—cristae fragmentation, mPTP activation, and ATP synthesis collapse—that selectively eliminates malignancy-permissive stromal niches^35,36^.

While bactericidal antibiotics broadly induce mitochondrial ROS^37,38^, cephalosporins uniquely weaponize this phenomenon through HMOX1-mediated iron-redox targeting. Unlike quinolones/aminoglycosides—which indiscriminately damage host cells via ROS—CUS exploits tumor cells’ inherent iron addiction and antioxidant deficits, achieving therapeutic specificity. This distinction is critical: murine models demonstrate cephalosporins’ tumor-selective cytotoxicity alongside systemic antioxidant upregulation, contrasting with the pan-tissue oxidative damage caused by other antibiotics^38^.

The HMOX1-ferroptosis axis thus represents both a vulnerability marker and therapeutic lever in inflammation-associated cancers. While our multimodal evidence positions HMOX1-driven ferroptosis as the dominant pathway, future investigations using HMOX1-knockout models will establish a definitive causal framework. This mechanistic paradigm not only recontextualizes cephalosporins as modulators of iron-redox biology but also provides a template for developing HMOX1-focused therapies against inflammation-associated malignancies.

## Conclusion

Our study redefines cephalosporin antibiotics as first-in-class therapeutics capable of intercepting inflammation-driven carcinogenesis through ferroptotic elimination of malignancy-permissive niches. While cephalosporins exhibit broad-spectrum anticancer activity, their therapeutic precision is exemplified by cefuroxime (CUS)—a β-lactam derivative whose tumor-selective cytotoxicity and safety profile stem from sidechain-driven HMOX1 induction rather than core β-lactam pharmacology. The HMOX1-ferroptosis axis, activated through iron-redox dysregulation (40–80 × transcriptional upregulation), suggests as the conserved mechanism licensing cephalosporins’ dual antimicrobial/anticancer action.

Despite this promise, dose-dependent attenuation at high therapeutic thresholds^39^ underscores the need for delivery optimization. We propose integrating ultrasound cavitation^40^ to enhance tumor-selective drug accumulation while mitigating systemic toxicity—a strategy synergistic with cephalosporins’ perioperative administration in curative-intent surgeries.

Collectively, our work exemplifies the power of drug repurposing paradigms in oncology. The CUS-driven HMOX1/ferroptosis axis not only provides a breakthrough strategy for intercepting inflammation-associated progression but also establishes cephalosporins as a clinically available candidate for precision prevention and therapy. Future studies delineating the evolutionary conservation of this mechanism across malignancies will further unlock the therapeutic potential of antimicrobial agents in cancer management.

## Supplementary Information

Supplementary Information 1.

Supplementary Information 2.

Supplementary Information 3.

Supplementary Information 4.

Supplementary Information 5.

Supplementary Information 6.

Supplementary Information 7.

Supplementary Information 8.

Supplementary Information 9.

Supplementary Information 10.

Supplementary Information 11.

Supplementary Information 12.

Supplementary Information 13.

Supplementary Information 14.

Supplementary Information 15.

Supplementary Information 16.

## Data Availability

The datasets during the current study available from the corresponding author on reasonable request. The mRNA-seq data is provided within the supplementary information files(the 12 text documents).
