# Correction: Repurposing cephalosporins as excellent anticancer agents and chemosensitizers for inflammation-driven cancer therapy

**DOI:** 10.1038/s41598-026-46038-3

**Published:** 2026-03-26

**Authors:** Nianqiu Liu, Weihan Cao, Jiefu Tang, Ruimei Dong, Juan Guo, Zhuang Luo, Qian Yao, Song Teng, Zhuoxuan Liang, Yuntao Yang, Menying Gu, Jie Zhou, Wenlin Chen, Hongmin Liang, Xiaoqiong He

**Affiliations:** 1 The School of Public Health, Kunming Medical University, Kunming, Yunnan China; 2 The Key Laboratory of Breast Surgery, The Third Affiliated Hospital of Kunming Medical University, Yunnan Cancer Hospital, Kunming, Yunnan China; 3 The Department of Ultrasound, The First Affiliated Hospital of Kunming Medical University, Kunming, Yunnan China; 4 The Department of Pulmonary and Critical Care Medicine, The First Affiliated Hospital of Kunming Medical University, Kunming, Yunnan China; 5 The Institute of Yunnan Tumor, The Third Affiliated Hospital of Kunming Medical University, Kunming, Yunnan China

Correction to: *Scientific Reports* 10.1038/s41598-025-25287-8, published online 21 November 2025

The original version of this Article contained an error in the name of author Hongmin Liang which was incorrectly given as Hongming Liang.

The original Article has been corrected.

