# Supplementary figures and images for: Repurposing cephalosporins as excellent anticancer agents and chemosensitizers for inflammation-driven cancer therapy
Source: Sci Rep. 2025 Nov 21;15:41380. doi: 10.1038/s41598-025-25287-8 (PMC12638924; doi:10.1038/s41598-025-25287-8)

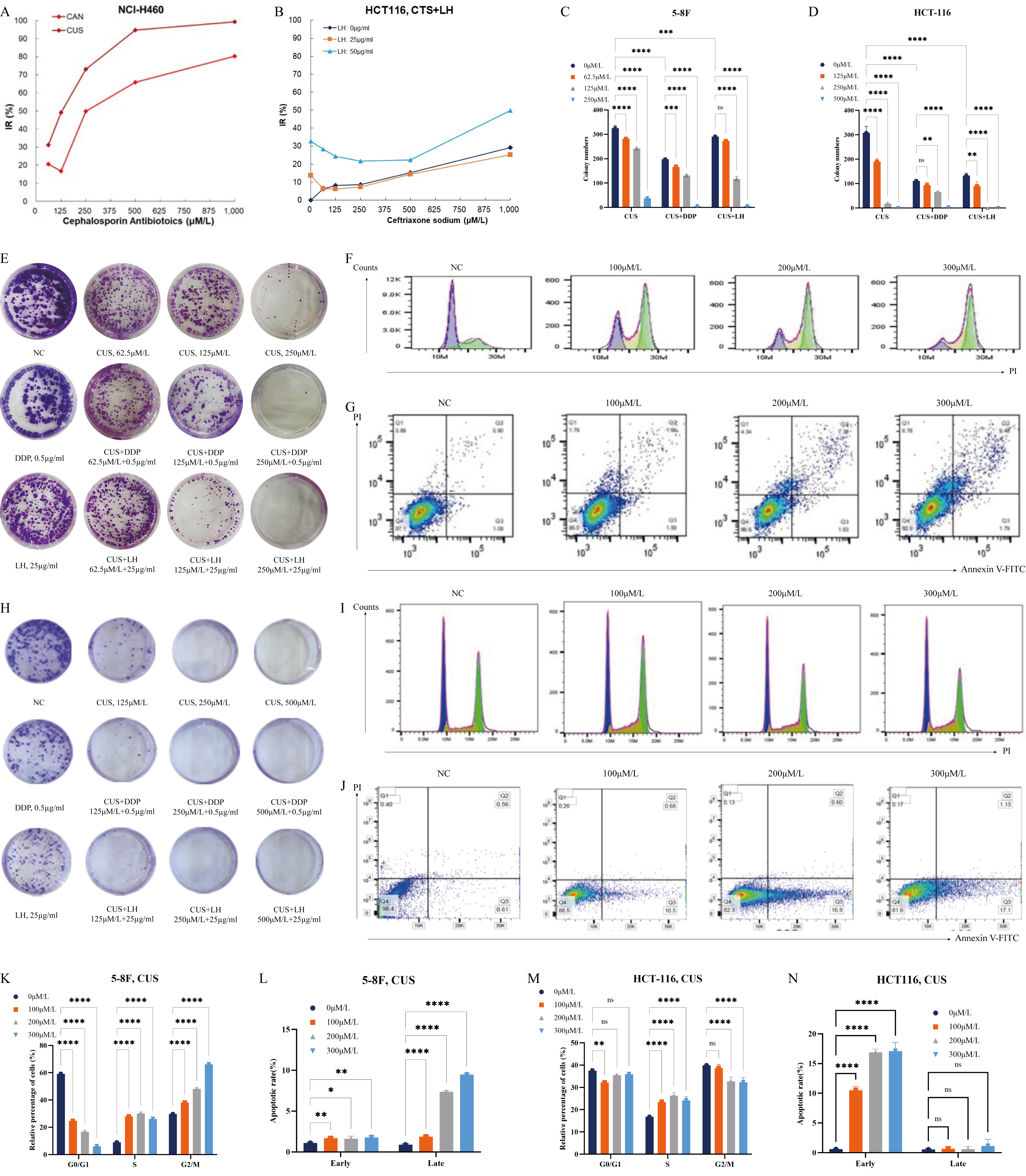

Supplement: Supplementary file 13 — Supplementary Information 13. [file 41598_2025_25287_MOESM13_ESM.tif]

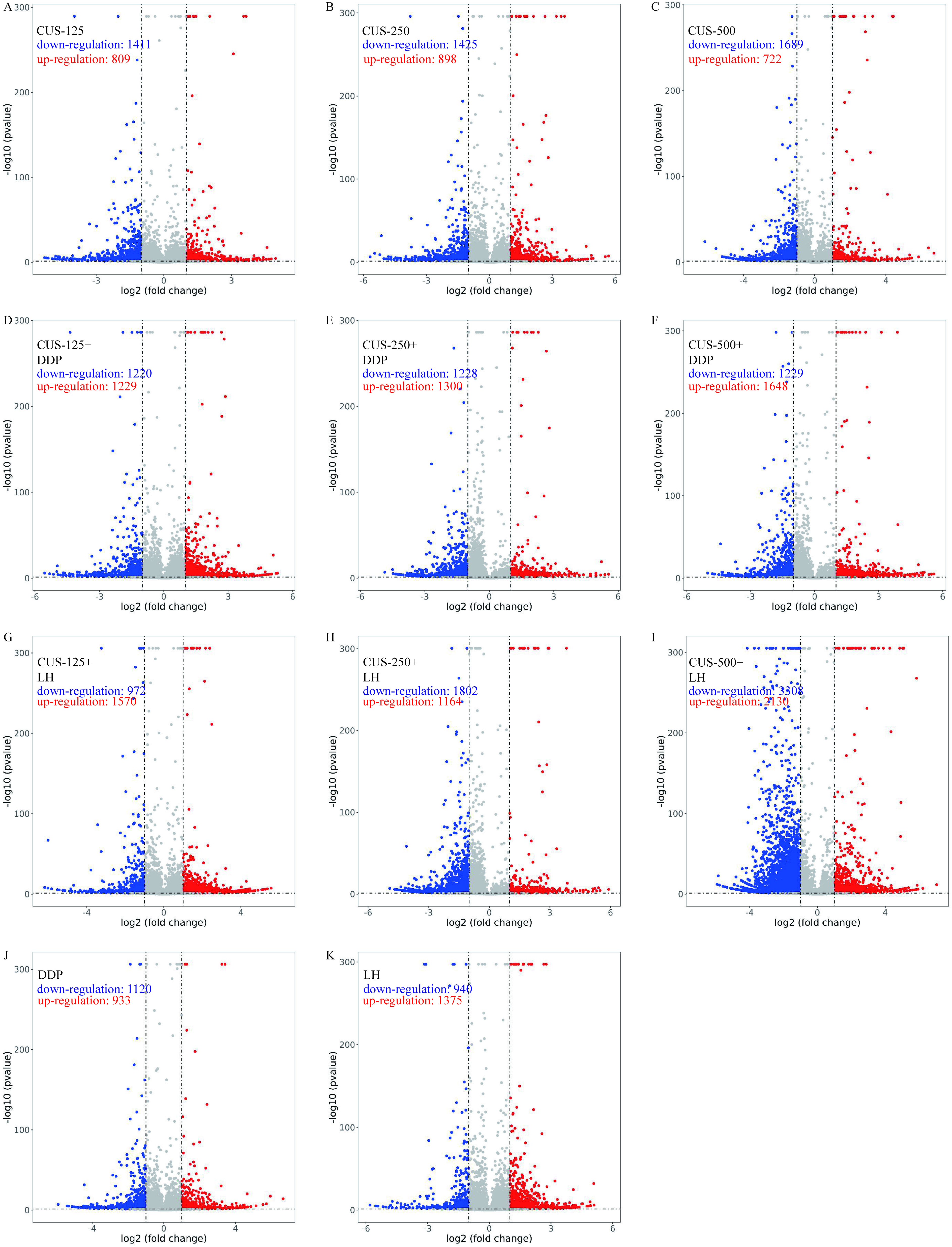

Supplement: Supplementary file 14 — Supplementary Information 14. [file 41598_2025_25287_MOESM14_ESM.tif]

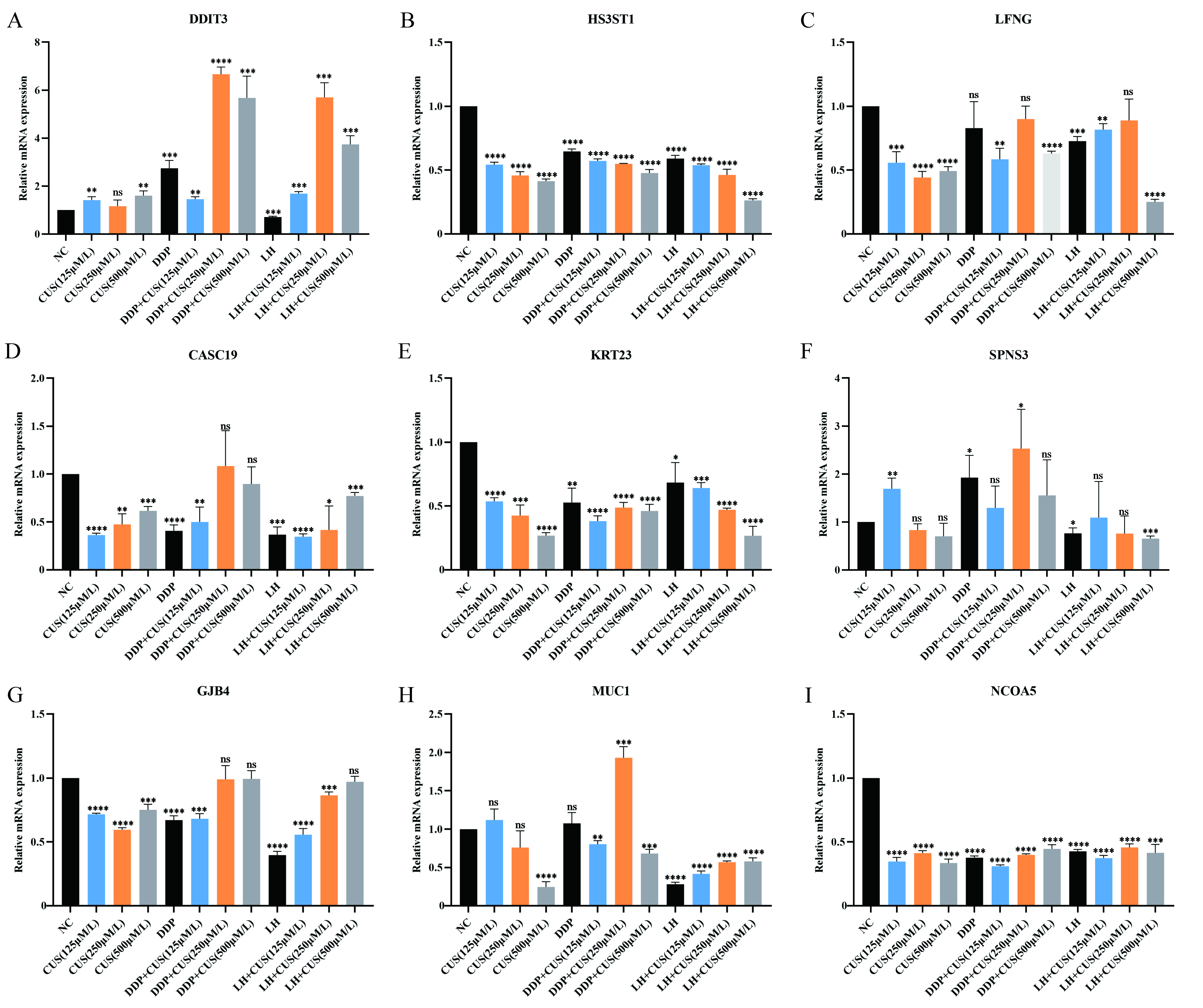

Supplement: Supplementary file 15 — Supplementary Information 15. [file 41598_2025_25287_MOESM15_ESM.tif]
