# Supplementary material for: Repurposing cephalosporins as excellent anticancer agents and chemosensitizers for inflammation-driven cancer therapy
Source: Sci Rep. 2025 Nov 21;15:41380. doi: 10.1038/s41598-025-25287-8 (PMC12638924; doi:10.1038/s41598-025-25287-8)
Supplement: Supplementary file 16 — Supplementary Information 16. [file 41598_2025_25287_MOESM16_ESM.docx]

**Supplementary Figure 1.** (A) IR of cell viability after CAN or CUS treatment of NCI-H460 . (B) IR of cell viability after CTS and LH treatment of in HCT-116. Colony formation numbers of 5-8F (C, E) and HCT-116 (D, H). Cell cycle changes of 5-8F (F, K) and HCT-116 (I, M). Apoptosis rate of 5-8F (G, L) and HCT-116 (J, N).

**Supplementary Figure 2.** Display differentially expressed genes after mRNA sequencing of different groups, with blue indicating down regulated genes and red indicating up regulated genes, (A) CUS group (0 vs 125µM/L). (B) CUS group (0 vs 250µM/L). (C) CUS group (0 vs 500µM/L). (D) CUS +DDP group (0+0.5μg/mL vs 125µM/L+0.5μg/mL). (E) CUS +DDP group (0+0.5μg/mL vs 250µM/L+0.5μg/mL). (F) CUS +DDP group (0+0.5μg/mL vs 500µM/L+0.5μg/mL). (G) CUS +LH group (0+25μg/mL vs 125µM/L+25μg/mL). (H) CUS +LH group (0+25μg/mL vs 250µM/L+25μg/mL). (I) CUS +LH group (0+25μg/mL vs 500µM/L+25μg/mL). (J) DDP group (0 vs 0.5μg/mL). (K) LH group (0 vs 25μg/mL).

**Supplementary Figure 3.** mRNA expression in different combination-groups. (A) DDIT3. (B) HS3ST1. (C) LENG. (D) CASC19. (E) KRT23. (F) SPNS3. (G) GJB4. (H) MUC1. (I) NCOA5.
